# Electrolyte Imbalance and Its Prognostic Impact on All-Cause Mortality in ICU Patients with Respiratory Failure

**DOI:** 10.3390/medicina61040642

**Published:** 2025-04-01

**Authors:** Oral Menteş, Deniz Çelik, Murat Yildiz, Abdullah Kahraman, Mustafa Özgür Cirik, Güler Eraslan Doğanay, Kerem Ensarioğlu, Munire Babayiğit, Derya Kizilgöz

**Affiliations:** 1 Department of Pulmonary Medicine, Faculty of Medicine, Health Sciences University, Atatürk Sanatorium Research Hospital, Ankara 06290, Turkey; omentes@live.com (O.M.); drmuratyildiz85@gmail.com (M.Y.); dr.ozgurr@hotmail.com (M.Ö.C.); gulerdoganay@hotmail.com (G.E.D.); kerem.ensarioglu@gmail.com (K.E.); mnroksuz@hotmail.com (M.B.); derya.ozaydin999@gmail.com (D.K.); 2 Department of Pulmonary Medicine, Faculty of Medicine, Alaaddin Keykubat University, Antalya 07425, Turkey; 3 Etlik City Hospital, Ankara 06170, Turkey; abdullahhero100@gmail.com

**Keywords:** respiratory failure, electrolyte imbalances, hyponatremia, hypernatremia, hypokalemia, hypocalcemia, ICU mortality

## Abstract

*Background and Objectives*: Chronic obstructive pulmonary disease (COPD) and acute respiratory failure are critical clinical conditions associated with high mortality rates in intensive care units (ICUs). Electrolyte imbalances are significant variables that may influence all-cause ICU mortality in this patient group. In this retrospective study, we aimed to investigate the relationships between the serum levels of sodium (Na^+^), chloride (Cl^−^), potassium (K^+^), calcium (Ca^2^^+^), and magnesium (Mg^2^^+^) and all-cause ICU mortality in patients admitted with respiratory failure. Additionally, we conducted a detailed mortality analysis on the basis of sodium quartiles and pathological absolute sodium thresholds to reveal their associations with ICU mortality from all causes. *Materials and Methods*: A total of 1109 patients were analyzed between January 2022 and January 2024. The electrolyte levels measured at ICU admission, demographic data, APACHE II and SOFA scores, arterial blood gas results, BUN and creatinine levels, need for noninvasive mechanical ventilation, length of ICU stay, and survival outcomes were assessed. Statistical analyses were performed via Kaplan—Meier survival analysis and the Cox regression method. *Results*: Our findings revealed that patients with low potassium and calcium levels had significantly higher mortality rates (*p* < 0.05). When sodium levels were divided into quartiles, mortality risk markedly increased in both the lowest (Q1) and highest (Q4) quartiles. Cox regression analysis revealed that the mortality risk in hyponatremic patients was 2.2 times greater than that in normonatremic patients (*p* = 0.005). In the hyponatremic group, the increased mortality risk was statistically borderline significant (*p* = 0.06). In the logistic regression analysis conducted to evaluate ICU mortality, which included all electrolyte levels and clinical scoring systems, higher APACHE II and SOFA scores were identified as significant risk factors for ICU mortality. Conversely, the presence of COPD was found to be relatively protective compared with other underlying causes of respiratory failure in terms of mortality. *Conclusions*: Electrolyte imbalances are important predictors of mortality in patients with respiratory failure. Sodium levels exhibit a “U-shaped” relationship with mortality, with hyponatremia emerging as a prominent risk factor. Careful assessment of electrolyte imbalances is crucial in the clinical management of these patients.

## 1. Introduction

Chronic obstructive pulmonary disease (COPD) and acute respiratory failure are among the serious clinical conditions associated with high mortality rates in intensive care units (ICUs). Electrolyte imbalances are significant variables that may influence mortality in this patient group. Sodium (Na^+^) and chloride (Cl^−^) levels are considered independent prognostic markers in critical illness processes [1,2].

Hypochloremia has been independently associated with mortality in critically ill patients. For example, a study conducted in ICU patients reported that hypochloremia significantly increased ICU mortality [1]. Similarly, in patients with acute kidney injury, chloride levels have a nonlinear relationship with mortality, with lower chloride levels being linked to an increased risk of death [2].

The prognostic impact of sodium is also crucial in this context. Both hyponatremia (low sodium levels) and hypernatremia (high sodium levels) have been identified as markers that increase mortality risk in various critical illness scenarios. In general, among medical patients presenting to the emergency department, both extremes of sodium levels have been shown to be associated with higher mortality rates [3].

Studies conducted in ICU settings on COPD patients have specifically evaluated the impact of electrolyte imbalances on mortality. For example, research on COPD patients undergoing mechanical ventilation due to acute respiratory failure has demonstrated a strong association between low sodium levels and high mortality rates [4]. Additionally, the prognostic significance of chloride has been investigated in conditions such as heart failure, where low chloride levels were found to increase mortality risk, whereas sodium alone did not fully explain this relationship [5].

Electrolyte imbalances play crucial roles not only in predicting mortality but also in guiding treatment processes. A study examining the use of chloride-rich solutions in ICU patients explored the impact of chloride level changes on patient outcomes [6]. Moreover, metabolic imbalances have been highlighted as key contributors to mortality in adult patients with cystic fibrosis and respiratory failure [7].

The literature underscores the independent effects of electrolyte imbalances, particularly serum chloride and sodium imbalances, on mortality in critically ill COPD patients. A recent study reported an “L-shaped” relationship between serum chloride levels and 90-day and 365-day mortality in critically ill COPD patients, with mortality decreasing as chloride levels increased to 102 mmol/L [8]. Similarly, during COPD exacerbations, sodium and potassium imbalances are frequently observed, significantly worsening patient outcomes [9].

In this context, investigating the impact of sodium and chloride imbalances on mortality in ICU patients with COPD and respiratory failure will contribute significantly to the literature and help clarify how these parameters can be utilized in clinical decision-making. In our study, we aimed to explore the relationship between sodium and chloride levels and all-cause mortality in a broad patient population with respiratory failure while also assessing their associations with disease severity and organ failure due to infection.

## 2. Materials and Methods

### 2.1. Ethical Approval and Study Design

Before commencing the study, compliance with the ethical principles outlined in the Declaration of Helsinki was declared, and ethical approval was obtained from the Clinical Research Ethics Committee of the University of Health Sciences, Ankara Atatürk Sanatorium Training and Research Hospital (approval number: 2024-BÇEK/228, date: 12 February 2025). Following this approval, all patients diagnosed with type 1 and type 2 respiratory failure and monitored in the secondary-level pulmonary intensive care units of Ankara Atatürk Sanatorium Training and Research Hospital between January 2022 and January 2024 were retrospectively screened. Before the use of patient data in this retrospective study (excluding radiological images and photographs), the presence of signed and complete informed consent forms in patient records was verified. Patients who had not signed the consent form or who refused to share their clinical data were excluded from the study. Informed consent forms were obtained from all patients whose data were retrospectively analyzed.

A total of 1211 records of patients with type 1 and type 2 respiratory failure from January 2022 to January 2024 were reviewed. Of these, 46 patients were excluded because of incomplete or unsigned informed consent forms. Additionally, 26 patients who died within the first 24 h of ICU admission and 22 patients who were transferred to another clinic within the first 24 h were also excluded. As a result, 1109 patients were included in the study.

#### 2.1.1. Inclusion Criteria

- Patients aged 18 years or older and diagnosed with type 1 or type 2 respiratory failure.

#### 2.1.2. Exclusion Criteria

- Patients younger than 18 years.
- Patients who died within the first 24 h after ICU admission or were transferred to another clinic.
- Patients with incomplete or unsigned informed consent forms.

Demographic characteristics such as age and sex were recorded for all included patients. In addition, blood samples obtained during the first days of ICU admission were analyzed for sodium and chloride levels, as well as other electrolytes, including magnesium, calcium, and potassium. Venous blood gas parameters, including pH, partial carbon dioxide pressure (pCO_2_), base excess (BE), and bicarbonate (HCO_3_), were recorded. Disease severity scores, including the acute physiology and chronic health evaluation (APACHE II) and sequential (or sepsis-related) organ failure assessment (SOFA) scores, were also documented. Additional parameters, such as length of ICU stay, survival duration, blood urea nitrogen (BUN) level, creatinine level, albumin level, and the need for noninvasive mechanical ventilation (NIV), were recorded.

### 2.2. Statistical Analysis

Statistical analyses were conducted via IBM SPSS Statistics Version 27. Categorical data are presented as n (%), whereas ordinal and nonnormally distributed numerical data are expressed as median and minimum–maximum values. For normally distributed numerical data, the mean and standard deviation (SD) were reported.

For categorical variables:

✓ If all the cells contained more than 5 patients, the chi-squared test was applied.
✓ If at least one cell contained fewer than 5 patients, Fisher’s exact test was used.

For numerical variables:

✓ Normally distributed data were analyzed via Student’s *t* test.
✓ Nonnormally distributed data were assessed via the Mann—Whitney U test.

The effect sizes of significantly different means in normally distributed numerical variables were reported as Cohen’s d values. Normality was evaluated through descriptive statistics, including the Kolmogorov—Smirnov and Shapiro—Wilk tests, skewness—kurtosis values, histograms, and outlier distributions.

For comparisons involving more than two categorical groups:

✓ If the numerical variables followed a normal distribution, one-way ANOVA was used.
✓ If the numerical variables did not follow a normal distribution, the Kruskal—Wallis H test was applied.

Kaplan—Meier analysis was used for survival analysis, whereas Cox regression analysis was performed to determine the hazard ratio (HR) for mortality risk. A 95% confidence interval (CI) was applied in all the statistical analyses, and a *p* value of <0.05 was considered to indicate statistical significance.

## 3. Results

Among the patients included in the study, 692 (62.4%) were male, and 417 (37.6%) were female. The median age of all patients was 71 years (22–99). The median age was 73 years (22–96) in females and 68 years (24–99) in males. The median APACHE II score was 15 (range: 3–34) for all patients, with a median score of 15 (5–34) in females and 15 (3–34) in males. The median SOFA score was 1 (range: 1–9) for all patients, 2 (1–7) for females, and 1 (1–9) for males (Table 1).

Among the patients diagnosed with respiratory failure, 858 (77.4%) had type 2 respiratory failure, whereas 251 (22.6%) had type 1 respiratory failure. A total of 738 patients (66.5%) had a COPD diagnosis, whereas 371 (33.5%) did not. Noninvasive mechanical ventilation (NIMV) was applied to 742 patients (66.9%), while 367 (33.1%) did not receive NIMV (Table 1).

At the end of the ICU follow-up, 85 patients (7.7%) had died (exitus) during their ICU stay. A total of 745 patients (67.2%) were discharged, 150 patients (13.5%) were transferred to the pulmonary disease clinic (wards), and 129 patients (11.6%) were transferred from the secondary-level ICU to a tertiary-level ICU for further management.

When the relationship between electrolyte levels and all-cause ICU mortality was analyzed, no significant differences were found in the median values of sodium, chloride, or magnesium between patients who survived and those who did not. However, potassium and calcium levels were significantly lower in patients who died in the ICU than in survivors. The effect size for potassium was calculated as Cohen’s d = 0.33 (95% CI [0.11, 0.55]), indicating a small to moderate effect size. Similarly, calcium levels were significantly lower in the ICU mortality group (Table 2).

Sodium, chloride, and magnesium electrolytes did not follow a normal distribution. Therefore, these electrolytes were ranked from the lowest to the highest values within the patient population. They were then divided into quartiles, with the lowest 25% of values classified as Q1 (0–25%), followed by Q2 (25–50%), Q3 (50–75%), and Q4 (75–100%). Each quartile was considered a subgroup, and Kaplan—Meier survival analysis and Cox regression analysis were performed separately for sodium, chloride, and magnesium to assess ICU mortality across these quartiles. Additionally, statistical comparisons were made to determine whether there were significant differences in mortality between the quartile groups of these electrolytes.

**Figure 1:**
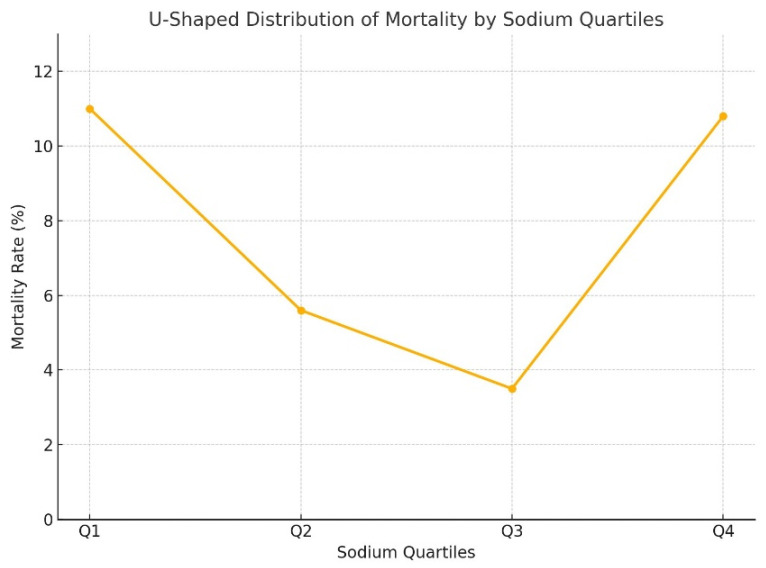
Mortality distribution according to the quartiles of sodium.

While no significant differences were found between the quartiles for magnesium and chloride in terms of mortality, the sodium levels in the Q1 and Q4 groups were significantly associated with higher mortality than were those in the Q2 and Q3 quartiles (*p* < 0.001) (Table 3). In other words, the relationship between sodium levels and mortality in our patient population followed a “U-shaped” pattern (Figure 1).

**Figure 2:**
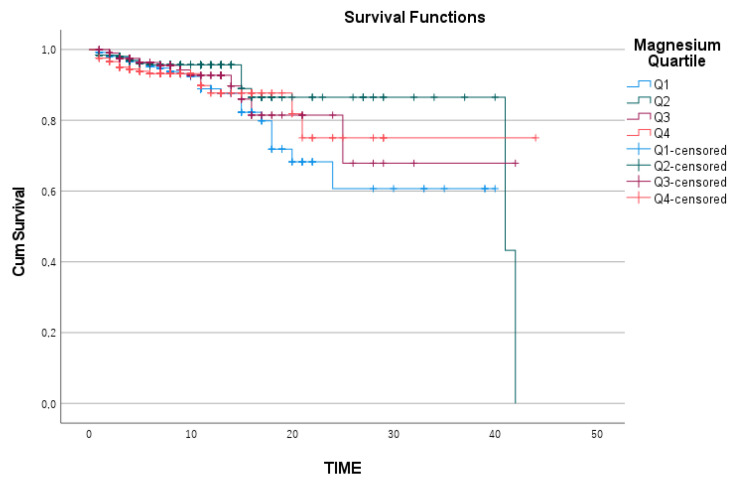
Magnesium Kaplan—Meier survival curve.

**Figure 3:**
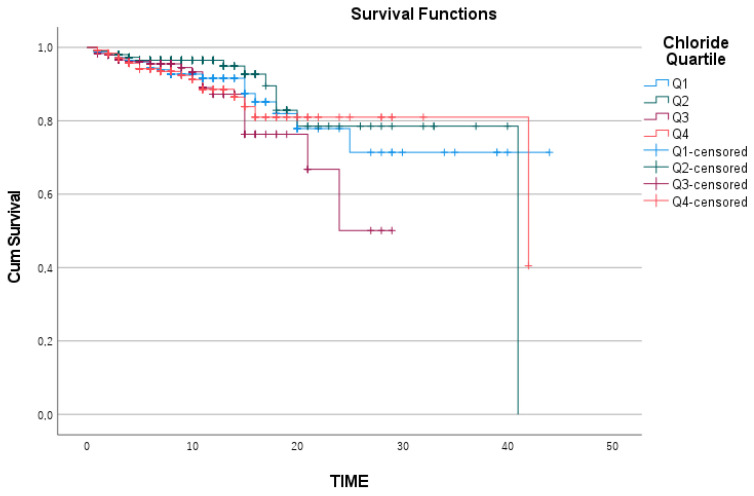
Chloride Kaplan—Meier survival curve.

When the Kaplan—Meier survival curves for the quartile subgroups of sodium, chloride, and magnesium were analyzed separately, the survival analysis for magnesium was statistically insignificant, with a log-rank value of 0.259 (Figure 2). Similarly, chloride levels were not significantly related to survival, with a log-rank value of 0.320 (Figure 3).

**Figure 4:**
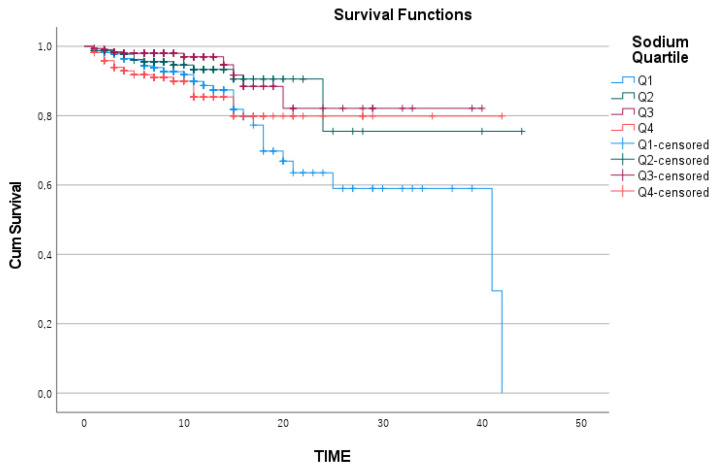
Sodium Kaplan—Meier survival curve.

However, for sodium levels, survival analysis between the quartiles revealed that the cumulative survival rate over time was significantly lower in the Q1 subgroup than in the other subgroups (log-rank = 0.002) (Figure 4). In the Cox regression analysis for ICU mortality, which included adjustments for the APACHE II and SOFA scores, the model was found to be statistically significant (*p* < 0.001) (Table 4).

**Figure 5:**
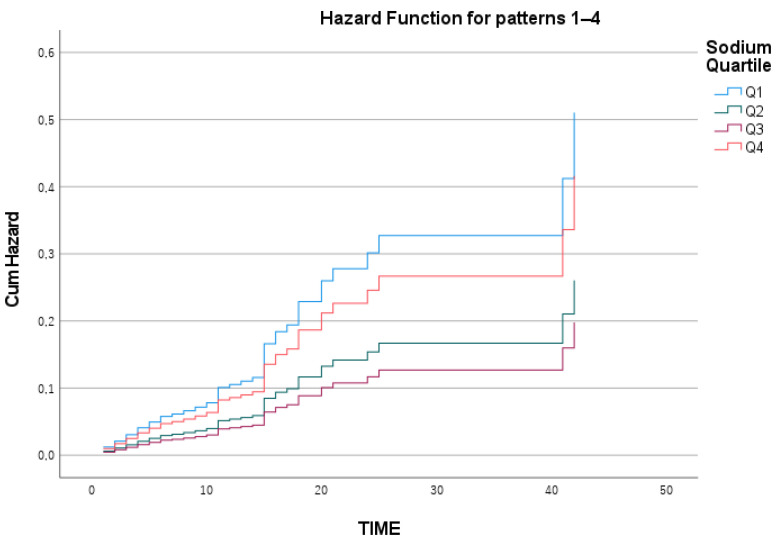
Sodium Cox regression hazard ratio plot.

**Figure 6:**
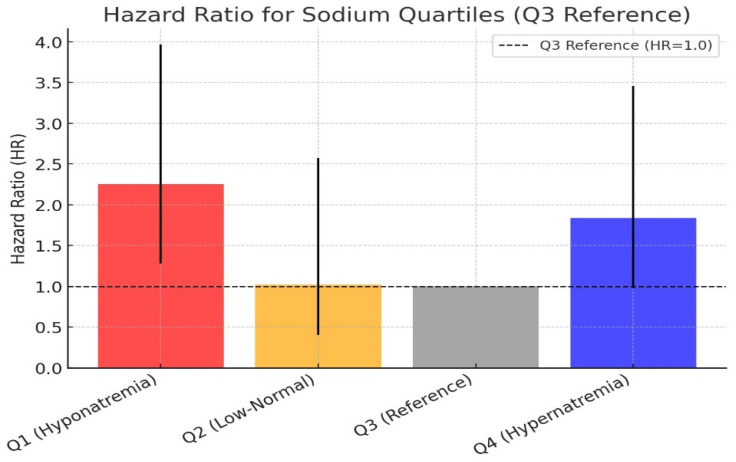
Hazard ratio values of sodium quartiles along with 95% confidence intervals.

The cumulative hazard ratio graph for the sodium quartiles is presented in Figure 5. Using the Q3 quartile (139–142 mEq/L) as the reference group, the hazard ratio for Q2 was calculated as 1.02, which was not statistically significant. However, the mortality risk in the Q1 group was approximately 2.2 times greater than that in the Q3 group (*p* = 0.005). In the Q4 group, the mortality risk was approximately 1.8 times greater than that in the other groups, but the result remained borderline statistically significant (*p* = 0.06). The hazard ratio comparison of the sodium quartile groups is shown in Figure 6.

Compared with those in the other quartiles, the albumin and potassium levels in the Q4 quartile were significantly lower (*p* = 0.003 and *p* < 0.001, respectively). When arterial blood gas parameters were examined, the pCO_2_ levels were significantly lower in Q1 and higher in Q4 than in Q2 and Q3 (*p* < 0.001). Similarly, bicarbonate levels were lower in Q1 and higher in Q4 than in Q2 and Q3 (*p* < 0.001).

BUN levels were also significantly greater in the Q4 group than in the other three groups (*p* = 0.003). The base excess values were lower in Q1, indicating metabolic acidosis, and higher in Q4 than in Q2 and Q3 (*p* < 0.001). While no significant differences were found in the APACHE II scores among the sodium quartiles, the SOFA scores were significantly higher in the Q4 group than in the other three groups (*p* = 0.001).

Another striking finding was that patients in the Q4 sodium quartile were significantly older than those in the other sodium quartiles were (*p* < 0.001). When the need for noninvasive mechanical ventilation (NIV) among sodium quartiles was evaluated, the Q1 group had a significantly lower requirement for NIV than the other groups did (*p* < 0.001).

Following the analysis based on the sodium quartiles of the patients included in our study, all patients were categorized into three groups according to internationally accepted serum sodium levels: hyponatremia (serum sodium < 135 mEq/L), normonatremia (135–145 mEq/L), and hypernatremia (serum sodium > 145 mEq/L). Statistical analyses conducted to evaluate the incidence of ICU mortality across these groups revealed a visibly higher rate of ICU mortality among patients with abnormal sodium levels (Table 5).

A statistically significant association was observed between sodium status (normonatremia, hyponatremia, hypernatremia) and ICU mortality (χ^2^ = 12.449, df = 2, *p* = 0.002) (Table 6).

Among patients with normonatremia, the mortality rate was 6.6% (60/908), whereas in hyponatremic patients, it increased to 9.9% (14/142), and in hypernatremic patients, it reached 18.6% (11/59). These findings indicate a progressively increasing mortality trend associated with dysnatremia, especially hypernatremia (Table 5).

Furthermore, the linear-by-linear association test (χ^2^ = 11.502, *p* < 0.001) supported the presence of a dose-dependent relationship between sodium imbalance severity and mortality risk (Table 6).

Effect size estimates, including Pearson’s *r* (0.102, *p* < 0.001) and Spearman correlation (0.090, *p* = 0.003), suggest a small but statistically significant positive correlation between dysnatremia and ICU mortality (Table 6).

A binary logistic regression analysis was performed via the enter method to assess the presence of ICU mortality on the basis of the variables found to be associated with mortality. Accordingly, age, APACHE II score, SOFA score, presence of COPD, type of respiratory failure (patients without COPD and those with type 1 respiratory failure were found to be at higher risk for ICU mortality), potassium, calcium, and the presence of hypo- and hypernatremia (compared with normonatremia) were included in the model for analysis.

The final model revealed that higher APACHE II and SOFA scores were significantly associated with increased odds of ICU mortality (*p* = 0.001 and *p* < 0.001, respectively). Additionally, the presence of COPD was identified as a strong protective factor (OR = 0.333, *p* < 0.001). Other variables, including age, sodium abnormalities, type of respiratory failure, potassium, and calcium, were not found to be statistically significant predictors in this model. The model demonstrated acceptable explanatory power, with a Nagelkerke R^2^ of 0.202 and a good fit, as indicated by the nonsignificant Hosmer and Lemeshow test (*p* = 0.390) (Table 7).

## 4. Discussion

In our study, when we reviewed all the electrolyte levels measured during the ICU stays of patients diagnosed with respiratory failure, we found that patients with lower potassium and calcium levels had higher mortality rates. A study conducted in 2023 on 104 patients with COPD exacerbation reported that hypokalemia was a risk factor for mortality. The same study also identified hyponatremia as a mortality risk factor [10]. Initially, our findings were interpreted as being consistent with the literature.

A study conducted in a surgical ICU identified hypocalcemia as a risk factor for respiratory failure; however, it did not specify the impact of hypocalcemia on mortality in respiratory failure patients. In our study, we determined that patients with low calcium levels in the respiratory failure group had higher mortality rates. This finding may indicate the presence of additional metabolic comorbidities in this patient population [11].

At first glance, sodium, chloride, and magnesium appeared to be insignificant in terms of mortality. However, considering that both high and low levels of these electrolytes might be clinically relevant, we performed statistical analyses by categorizing them into quartiles. For sodium, we obtained striking results—both high and low sodium levels were associated with all-cause ICU mortality.

A similar relationship has been previously reported for chloride levels in COPD patients, where chloride levels were linked to 90-day and 365-day all-cause mortality. In a Cox regression analysis using 102 mmol/L as a reference point, the hazard ratio for hypochloremic levels increased to 3, decreased below 1 at slightly higher chloride levels, and then rose again beyond 102 mmol/L, forming an “L-shaped” hazard ratio graph rather than a linear correlation [8]. In our study, sodium followed a “U-shaped” hazard ratio curve, with the hyponatremic side of the “U” being more extended and showing higher hazard ratios.

Thongprayoon et al. examined sodium levels at hospital discharge and reported that both hyponatremia and hypernatremia were associated with one-year mortality risk. Their study also described a “U-shaped” relationship between sodium intake and mortality [12]. Interestingly, a study on cardiovascular diseases demonstrated a similar “U-shaped” relationship, not with serum sodium levels but with sodium intake, indicating poor health outcomes [13].

To account for the possibility that certain conditions specific to respiratory failure patients might increase mortality and falsely attribute this effect to sodium quartiles, we compared sodium quartiles on the basis of APACHE II and SOFA scores. Statistical analyses revealed notable findings for the hypernatremic Q4 group. Compared with the other groups, this group had higher partial pCO_2_ levels, BUN levels, and SOFA scores and consisted of older patients. However, for the Q1 group, the only significant finding was low pH, while pCO_2_ levels were significantly lower, indicating metabolic acidosis. Moreover, despite the increased mortality risk in the sodium Q1 group, the lower need for NIV than in the other groups emerged as a striking finding. In the literature, the need for mechanical ventilation has been associated with increased mortality in patients admitted to the ICU with type 1 respiratory failure and monitored with a diagnosis of acute idiopathic pulmonary fibrosis exacerbation [14]. Considering this information, the fact that we identified a lower attributable mortality risk factor in hyponatremic patients with respiratory failure supports the notion that hyponatremia is an independent prognostic factor.

A study investigating independent risk factors for hyponatremia in COPD patients identified pneumonia, hypomagnesemia, and metabolic acidosis as closely related to hyponatremia [15]. Additionally, numerous studies in the literature have reported that hyponatremia serves as a poor prognostic indicator in pediatric patients with lower respiratory tract infections [16,17,18]. Hyponatremia is also frequently observed in lung malignancies, particularly in small-cell lung cancer and, to a lesser extent, in nonsmall-cell lung cancer, where it presents as a paraneoplastic syndrome and is considered a poor prognostic marker [19,20].

As observed in various pulmonary pathologies, hyponatremia frequently occurs and is associated with worse disease outcomes. In our study, we found that patients with the lowest sodium levels had approximately 2.2 times higher mortality rates than did those with normal sodium levels. Although the hypernatremic group also exhibited a borderline significant increase in mortality risk, these patients had multiple additional risk factors that could contribute to mortality. Increased SOFA scores indicate sepsis, whereas elevated renal function markers suggest acute kidney injury, a well-known predictor of mortality and morbidity. Furthermore, the hypernatremic group consisted of older patients, further contributing to their poor outcomes.

These findings suggest that rather than hypernatremia itself being the direct cause of increased mortality, it may be a consequence of underlying clinical conditions such as hypovolemic hypernatremia, prerenal acute kidney injury, and infections. In contrast, hyponatremia has diverse and complex etiologies, all of which can serve as mortality predictors. However, persistent hypernatremia should not be overlooked, as it has been associated with prolonged ICU stays, increased ICU mortality, and increased postdischarge mortality [21].

Beyond the analysis based on sodium quartiles, when we categorized the study population into hyponatremia (<135 mEq/L), normonatremia (135–145 mEq/L), and hypernatremia (>145 mEq/L) groups according to absolute sodium values, we obtained similar results; however, we observed that pathological hypernatremia was more strongly associated with ICU mortality. In the literature, a study conducted in Australia that included 55,255 patients from 12 intensive care units identified all hypernatremia levels above 145 mEq/L as independent risk factors for 30-day ICU mortality after adjusting for other variables. Furthermore, this study revealed that ICU-acquired hypernatremia was associated with fever, invasive ventilation, higher APACHE III scores, and prior diuretic use before the onset of hypernatremia [22].

### Study Limitations

In this study, we evaluated the impact of electrolyte levels on all-cause ICU mortality in patients with respiratory failure. However, ICU physicians are well aware that ICU mortality can result from a wide range of factors. In some cases, acute coronary syndrome or cerebrovascular events, which may develop independently of the patient’s primary clinical condition, can also contribute to mortality in ICU patients. Large prospective studies that comprehensively investigate the causes of mortality would help address this major limitation of our study.

Additionally, the term “respiratory failure” describes a broad range of clinical conditions rather than a specific disease group. More specific studies focusing on individual diseases could provide more valuable contributions to the literature.

## 5. Conclusions

The importance of serum electrolyte levels in the ICU is well recognized by physicians. However, electrolyte imbalances are so frequently encountered in ICU settings that electrolyte and fluid replacement therapies have become a routine part of daily practice for ICU physicians. Similar to the phenomenon of “ICU alarm fatigue” in response to invasive mechanical ventilator alarms, clinicians may become desensitized to electrolyte imbalances, leading to replacement therapies that are prescribed almost automatically without much consideration.

In many cases, ICU physicians do not reflect on the prognostic implications of these imbalances or hesitate to order additional diagnostic tests for differential diagnosis. However, electrolyte imbalances remain among the most common challenges ICU patients face, regardless of their reason for admission. Therefore, we believe that each electrolyte should be assessed individually to predict disease prognosis effectively and guide appropriate treatment interventions.

## Figures and Tables

**Table 1:** Baseline demographic and clinical characteristics of the study population.

Variable	n (%), Mean ± SD, Median (Min–Max)	
Gender	417 female (37.6%)	692 male (62.4%)
Presence of COPD	738 yes (66.5%)	371 no (33.5%)
Age ≥ 65 years	756 yes (68.2%)	353 no (31.8%)
Use of NIMV	742 yes (66.9%)	367 no (33.1%)
Respiratory failure	858 type 2 (77.4%)	251 type 1 (22.6%)
SOFA score	1.84 ± 1.11	2 (1–9)
APACHE II score	15.08 ± 4.16	15 (3–34)
Age	69.42 ± 11.68	70 (22–99)

COPD: chronic obstructive pulmonary disease; NIMV: noninvasive mechanical ventilation; SD: standard deviation; SOFA: sequential organ failure assessment; APACHE II: acute physiology and chronic health evaluation II.

**Table 2:** The impact of electrolyte levels on mortality.

Parameters	ICU Mortality (+) Mean ± SD Median (Min–Max)	ICU Mortality (−) Mean ± SD Median (Min–Max)	*p*-Value
Sodium (mEq/L)	138 (119–157)	139 (110–152)	0.445 ^a^
Chloride (mEq/L)	99 (69–114)	98 (75–114)	0.589 ^a^
Potassium (mEq/L)	4.18 ± 0.76	4.39 ± 0.65	0.004 *^,b^
Magnesium (mg/dl)	1.9 (0.9–3.3)	2 (0.6–3.8)	0.512 ^a^
Calcium (mg/dl)	8.3 (6.3–12.7)	8.7 (5.3–12.2)	<0.001 *^,a^

ICU: intensive care unit, ^a^ Mann—Whitney U test, ^b^ Student’s *t* test, * significant values, CI: 95%.

**Table 3:** Comparison of the quartiles of sodium, chloride, and magnesium electrolytes in terms of mortality.

Parameters	ICU Mortality (+) n (%)	ICU Mortality (−) n (%)	*p*-Value
Sodium Q1	37 (11%)	299 (89%)	<0.001 *^,c^
Sodium Q2	13 (5.6%)	219 (94.4%)	
Sodium Q3	11 (3.5%)	307 (96.5%)	
Sodium Q4	24 (10.8%)	199 (89.2%)	
Chloride Q1	25 (8.5%)	270 (91.5%)	0.398 ^c^
Chloride Q2	17 (5.5%)	294 (94.5%)	
Chloride Q3	21 (8.6%)	222 (91.4%)	
Chloride Q4	22 (8.5%)	238 (91.5%)	
Magnesium Q1	35 (9.4%)	338 (90.6%)	0.265 ^c^
Magnesium Q2	18 (5.8%)	295 (94.2%)	
Magnesium Q3	14 (6.5%)	203 (93.5%)	
Magnesium Q4	18 (8.7%)	188 (91.3%)	

ICU: intensive care unit, ^c^ chi-squared test, * significant values, CI: 95%.

**Table 4:** Sodium quartiles along with APACHE II and SOFA scores in Cox regression analysis data.

Variable	B	SE	Wald	df	Sig. (*p*)	HR (Exp(B))	95% CI (Lower–Upper)
APACHE II	0.1	0.024	17.082	1	<0.001 *	1.105	(1.054–1.159)
SOFA	0.36	0.079	20.712	1	<0.001 *	1.433	(1.227–1.674)
Q3 (Reference)			9.611	3	0.022 *		
Q1 (Low Sodium)	0.812	0.289	7.913	1	0.005 *	2.252	(1.279–3.964)
Q2 (Mid–Low Sodium)	0.018	0.473	0.001	1	0.970	1.018	(0.403–2.573)
Q4 (High Sodium)	0.607	0.323	3.542	1	0.060	1.836	(0.975–3.456)

APACHE II: acute physiology and chronic health evaluation, B: beta coefficient, CI: confidence interval, HR: hazard ratio, SE: standard error, SOFA: sequential organ failure assessment, Wald: Wald test, df: degrees of freedom, * significant values.

**Table 5:** Associations between sodium imbalance and ICU mortality.

Sodium Status	Alive (n)	Deceased (n)	Total (n)	Mortality Rate (%)	*p*-Value (Test Used)
Normonatremia	848	60	908	6.6%	*p* = 0.002 (Chi-square, Monte Carlo)
Hyponatremia	128	14	142	9.9%	
Hypernatremia	48	11	59	18.6%	

**Table 6:** Detailed statistical tests analyzing the interrelationships of sodium abnormalities.

Statistical Test	Value	df	Asymptotic *p*-Value	Monte Carlo *p* Value (2-Sided)	95% CI (Monte Carlo)
Pearson chi-squared	12.449	2	0.002	0.002	[0.000–0.004]
Likelihood ratio	9.793	2	0.007	0.014	[0.007–0.020]
Fisher–Freeman–Halton exact test	10.675	-	-	0.004	[0.000–0.007]
Linear-by-linear association	11.502	1	<0.001	<0.001	[0.000–0.003]

df: degrees of freedom.

**Table 7:** Logistic regression analysis for ICU mortality.

Variable	B	SE	Wald	df	*p*-Value	Exp(B) (OR)
Age	0.008	0.011	0.588	1	0.443	1.008
Hyponatremia vs. normonatremia	0.3	0.337	0.79	1	0.374	1.349
Hypernatremia vs. normonatremia	0.454	0.428	1.128	1	0.288	1.575
APACHE II	0.095	0.028	11.316	1	0.001	1.099
SOFA	0.335	0.092	13.294	1	0.0	1.398
Respiratory failure type (type 2 present)	−0.293	0.27	1.178	1	0.278	0.746
COPD (present)	−1.1	0.266	17.084	1	0.0	0.333
Potassium	−0.157	0.185	0.717	1	0.397	0.855
Calcium	−0.177	0.161	1.21	1	0.271	0.837
Constant	−2.891	1.691	2.923	1	0.087	0.056

APACHE II: acute physiology and chronic health evaluation II, SOFA: sequential organ failure assessment, COPD: chronic obstructive pulmonary disease, OR: odds ratio, SE: standard error.

## Data Availability

Due to legal obligations, the data from our study cannot be shared publicly. However, upon reasonable request to the corresponding author, the data may be made available for scientific purposes in full compliance with personal data protection regulations (with all personal identifiers anonymized).
